# Antibodies against Lagos Bat Virus in Megachiroptera from West Africa

**DOI:** 10.3201/eid1406.071421

**Published:** 2008-06

**Authors:** David T.S. Hayman, Anthony R. Fooks, Daniel Horton, Richard Suu-Ire, Andrew C. Breed, Andrew A. Cunningham, James L.N. Wood

**Affiliations:** * University of Cambridge, Cambridge, UK; † Veterinary Laboratories Agency Weybridge, Surrey, UK; ‡ Institute of Zoology, London, UK; § Wildlife Division of the Ghana Forestry Commission, Accra, Ghana; ¶ University of Queensland, Brisbane, Queensland, Australia

**Keywords:** Lagos Bat Virus, rabies, megachiroptera, bat, Lyssavirus, dispatch

## Abstract

To investigate the presence of Lagos bat virus (LBV)–specific antibodies in megachiroptera from West Africa, we conducted fluorescent antibody virus neutralization tests. Neutralizing antibodies were detected in *Eidolon helvum* (37%), *Epomophorus gambianus* (3%), and *Epomops buettikoferi* (33%, 2/6) from Ghana. These findings confirm the presence of LBV in West Africa.

Bats host a range of lyssaviruses, depending on their species and locality. The genus *Lyssavirus* is differentiated into 7 genetically divergent genotypes: classical *rabies virus* (genotype 1), *Lagos bat virus* (LBV; genotype 2), *Mokola virus* (MOKV; genotype 3), *Duvenhage virus* (genotype 4), *European bat lyssavirus* (genotypes 5 and 6), and *Australian bat lyssavirus* (genotype 7) (*1*). All but MOKV have been isolated from bats.

LBV and MOKV are each distributed in Africa and are members of phylogroup 2 within the genus *Lyssavirus* (*1*). Because LBV isolates (*2*) from African bats are increasing, our goal was to determine the prevalence of virus neutralizing antibodies against LBV in bat populations in West Africa.

## The Study

Bats were sampled in January and May 2007 at 6 sites in Ghana: the center of Accra (urban habitat); the wooded outskirts of Accra (savannah habitat); and forested habitats at Pra, Kibi, Adoagyiri, and Oyibi (a plantation with woodland/forest border). Bats were captured by using 6–18-m mist nets; roosting *Eidolon helvum* were captured by using nets on poles. A sample size of 59 would provide 95% confidence of finding at least 1 LBV-seropositive bat in a large population (>5,000), given a seroprevalence of 5% and assuming random sampling (*3*). Species were identified by using a dichotomous key (*4*). Captured bats were manually restrained and anesthetized by intravenous injection; ≈0.2–1.0 mL of blood was collected from the propatagial vein before the bat was released. Blood was centrifuged in the field at ambient temperature at 3,000 rpm for 15 min. Serum was heat treated at 56°C for 30 min and frozen at –70°C.

Two species, *Epomophorus gambianus* and *E. helvum,* were caught in sufficient numbers (117 and 66, respectively) for reasonable inferences to be made about LBV seroprevalence rates (Table). A standard approach was used to calculate 95% confidence intervals (CIs) for seroprevalence (*3*). Because of the relatively short distances between study sites and the likelihood of bats mixing between these sites, bats of each species were considered to belong to single populations. All but 3 *E. helvum* were derived from a colony in Accra, whereas *E. gambianus* were derived from all habitat types.

**Table:** Bat species and their respective seroprevalence rates against phylogroups 1and 2 lyssaviruses, Ghana, 2007*

Species	Habitat	No. caught	% Adults tested for LBV antibodies	Seroprevalence, % (95% CI, no. tested)	
				CVS rabies virus	LBV
*Epomophorus gambianus*	Savannah†	117	61	0 (49)	3 (0–7, 91)
*Eidolon helvum*	Urban‡	66	95	0 (57)	37 (24–49, 57)
*Epomops franqueti*	Forest‡	30	77	0 (3)	0 (31)
*Epomops buettikoferi*	Forest‡	9	83	0 (5)	30 (0–70, 6)
*Hypsignathus monstrosus*	Forest‡	18	56	0 (1)	0 (5)
*Nanonycteris veldkampii*	Forest‡	5	100	NT	0 (4)

*Phylogroup 1 contains challenge virus standard (CVS), genotype 1; phylogroup 2 contains Lagos bat virus (LBV), genotype 2; CI, confidence interval; NT, not tested. †*E. gambianus* was caught in all habitats, including at the city colony and in plantations. ‡A small number of these species were caught in plantations.

Bat serum samples were tested for virus neutralizing antibody against classical rabies virus (challenge virus standard) by using a standard fluorescent antibody virus neutralization (FAVN) test (*5*). Antibodies to LBV were measured by using a modified FAVN test (*6*). Because positive bat antiserum from naturally infected bats was not available, for positive controls we used human rabies immunoglobulin, LBV-positive rabbit serum, and serum from mice vaccinated with human diploid cell vaccine. Negative controls were negative rabbit and mouse serum. All samples were analyzed in duplicate and serially diluted by using a 3-fold series (representing reciprocal titers of 9, 27, 81, and 243–19,683) (*6*).

**Figure 1:**
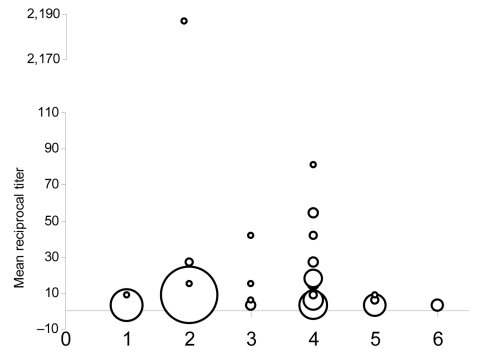
Antibody titers to Lagos bat virus (LBV) in 6 species of fruit bat in Ghana. An LBV-specific modified fluorescent antibody neutralization test was used to determine the level of antibody in bats; it used two 3-fold serial dilutions and derived a mean dilution at which the bats neutralized LBV. Bats with mean titers >9 were considered positive. The circle size represents the number of bats tested. 1, *Epomops franqueti;* 2, *Epomophorus gambianus*; 3, *Epomops buettikoferi*; 4, *Eidolon helvum*; 5, *Hypsignathus monstrosus*; 6, *Nanonycteris veldkampii.*

The modified FAVN test requires a cut-off threshold, which in prior bat lyssavirus studies has been a titer of 27, to avoid false-positive results (*6**,**7*). The first 121 samples collected were tested against the challenge virus standard; no tested bat was seropositive at 1:3 dilutions. A mean titer >9 was considered positive for LBV (Figure 1; [*8*]).

Levels of specific virus neutralizing antibodies against LBV were higher in *E. helvum* (seroprevalence 37%, 95% CI 24%–49%) than in *E. gambianus* (3%, 95% CI 0%–7%). Of 6 *Epomops buettikoferi*, 2 were seropositive (30%, 95% CI 0%–70%). No sex differences in *E. helvum* seroprevalence were evident (χ^2^ 1.0, p>0.9).

Because of the high level of seropositivity in *E. helvum*, we attempted to determine a possible case reproduction rate (R_0_) for LBV infection in this species by using the equation R_0_ = 1/x*, where x* = proportion of susceptible hosts in a population (*9*). We assumed that infection with each virus within the bat populations is endemic, stable, and randomly dispersed; that all seropositive animals have lifelong immunity that is detectable serologically; and that seropositivity is to 1 virus. On the basis of these assumptions, R_0_ = 1.6 (95% CI 1.3–2.0).

## Conclusions

We found antibodies against LBV in healthy *E. helvum* bats in Ghana. Previous studies have suggested that healthy bats develop antibodies to other lyssavirus infections (*7**,**10**,**11*), which may reflect past exposure, rather than survival from clinical disease. LBV likely co-evolved with its natural megachiropteran host until a genetic stasis had been reached in which the virus–host relationship was in equilibrium. This situation would result in high seroprevalence rates after a wave of virus circulation in a colony. Nine seropositive bats (8 *E. helvum,* 1 *E. buettikoferi*) were apparently healthy pregnant females. These results support theories that lyssaviruses are endemic within specific bat populations, that they may not cause high mortality rates, that exposure rates of LBV between megachiroptera in Old World African bats are high, and that bats may breed successfully after LBV exposure (*7**,**8*). The number of high reciprocal titers against LBV (Figure 1) and the history of LBV isolation in *E. helvum* suggest that LBV circulates in megachiroptera in Ghana. However, further work is needed to determine the specific phylogroup 2 virus and its prevalence within specific bat populations.

No previous estimate of R_0_ for genotype 2 *Lyssavirus* has been calculated, and although anamnesis may lead to no detectable antibodies in bats with immunity and a consequent underestimate of R_0_, this value indicates the potential R_0_ and is comparable to values previously estimated for lyssavirus infections in bats (*7**,**11*). More detailed analysis relating to age-specific infection and survival rates or mode of transmission was precluded by the difficulty in determining the age of adult bats, the lack of juveniles in the sample, and the cross-sectional sample used.

**Figure 2:**
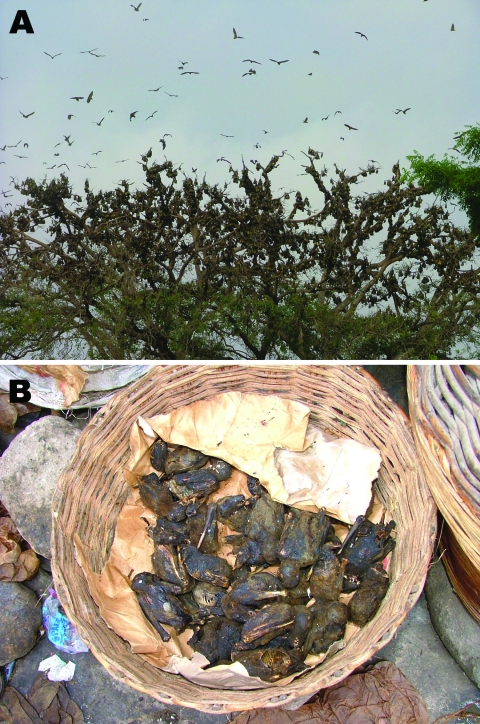
A) Density of a typical *Eidolon helvum* roost in the Accra colony. B) *E. helvum* as bushmeat in an Accra market.

The underlying cause of the difference in seroprevalence between *E. gambianus* and *E. helvum* with respect to LBV infection is unclear. Possible explanations include differential susceptibilities to infection; virus–host adaptation; different contact with the virus, including a recent epidemic in the *E. helvum* colony; or different population ecology. *E. helvum* resides in high-density populations (hundreds of thousands) (Figure 2, **panel A**) and migrates annually, compared with *E. gambianus*, which resides in less dense colonies of tens or hundreds (*4*). *E. helvum* commonly forms large colonies in African cities in close proximity to humans and domestic animals and is a food source in West Africa (Figure 2, **panel B**).

No investigations into infections of humans were made during these investigations, but lyssavirus infections in humans in Africa are underdiagnosed (*12*). Despite reduced pathogenicity of LBV in the laboratory, it has been isolated from dogs, cats, and a mongoose (*2*). Conversely, MOKV has caused a fatal case of encephalitis in a human (*1*). LBV and MOKV each have a substitution in the R333 glycoprotein residue (*1*). Although it is not the only protein to determine the pathogenicity of LBV, the R333 substitution still remains an important marker of rabies pathogenicity. In conclusion, the high seroprevalence to LBV in this population may pose a substantial public health risk because *E. helvum* is widely distributed in Africa and a food source in West Africa.
